# Economic and cost considerations of delivering and using mobile X‐ray services in residential aged care facilities: A qualitative study

**DOI:** 10.1111/ajag.13228

**Published:** 2023-07-30

**Authors:** Joanne Dollard, Jane Edwards, Lalit Yadav, Virginie Gaget, David Tivey, Maria C. Inacio, Guy J. Maddern, Renuka Visvanathan

**Affiliations:** ^1^ Adelaide Geriatrics Training and Research with Aged Care (GTRAC) Centre, Adelaide Medical School, Faculty of Health and Medical Sciences University of Adelaide Adelaide South Australia Australia; ^2^ Basil Hetzel Institute for Translational Health Research The Queen Elizabeth Hospital, Central Adelaide Local Health Network Adelaide South Australia Australia; ^3^ Discipline of Surgery, The University of Adelaide The Queen Elizabeth Hospital Adelaide South Australia Australia; ^4^ Royal Australasian College of Surgeons Adelaide South Australia Australia; ^5^ Registry of Senior Australians South Australian Health and Medical Research Institute Adelaide South Australia Australia; ^6^ Allied Health and Human Movement University of South Australia Adelaide South Australia Australia; ^7^ Aged and Extended Care Services The Queen Elizabeth Hospital, Central Adelaide Local Health Network Adelaide South Australia Australia

**Keywords:** mobile health units/economics, mobile health units, nursing homes, qualitative research, X‐rays

## Abstract

**Objective:**

To describe the economic and cost considerations of mobile X‐ray services (MXS) in residential aged care facilities (RACFs), according to stakeholders (involved in residents' healthcare), residents living in RACFs and informal carers (ICs) of residents.

**Methods:**

Semistructured interviews were conducted with 20 residents and 27 ICs recruited from six RACFs across metropolitan Adelaide (South Australia, Australia), and 22 stakeholders, on their perspectives of using MXS in RACFs. Data relating to economic and cost considerations were extracted and analysed using thematic analysis.

**Results:**

Residents' mean age was 85 years, 60% were women and 40% had experienced an MXS in the last 12 months. Most ICs were daughters (70%) and wives (11%) and 30% had a family member who had experienced an MXS in the last 12 months. Stakeholders included RACF staff, GPs, a hospital avoidance program clinician, paramedics, emergency department clinicians, MXS radiographers and manager, and a radiologist. Four themes were presented: (1) business considerations, where private providers found it necessary to charge residents a co‐payment to deliver MXS; (2) cost and payment process as a potential barrier to using MXS, with varied willingness and ability to pay for an MXS co‐payment, and equity concerns; (3) overcoming cost and payment barriers, with staff and consumers sometimes using strategies to overcome cost barriers; and (4) perceived cost benefits of MXS to the healthcare system, residents and ICs.

**Conclusions:**

Mobile X‐ray services providers charge residents an upfront co‐payment for business viability, which can be a barrier to some residents wishing to access MXS.

**Policy Impact**

Residents' willingness and ability to pay co‐payments to receive an MXS within RACFs could be a barrier to access and may contribute to inequities in care, impacting health outcomes. This presents an opportunity for policymakers to ensure that appropriate government subsidisation is provided to ensure equity and to improve access to quality in care.

## 1 INTRODUCTION

Some older people require ongoing care and support in residential aged care facilities (RACFs). Between 2020 and 2021, 243,117 Australians permanently resided in RACFs,^1^ and by 2050, it is estimated that there could be more than 1.5 million residents.^2^ Residential aged care facilities manage frail residents, who could benefit from avoiding hospitalisations, which could be done by receiving healthcare‐in‐place for select health conditions. Hospital avoidance interventions are of major interest to health‐care systems given that residents are major consumers of hospital services, comprising one in 14 hospital overnight stays.^3^ By providing health care, such as mobile X‐ray services (MXS) in RACFs, some hospital transfers could potentially be avoided, enabling residents to be supported in a familiar supportive environment.^4^ A 2015 evaluation of MXS delivered by one Australian hospital to residents in RACFs demonstrated a 12% reduction in emergency department (ED) presentations.^5^ Our recent qualitative research from the perspective of residents^6^ and informal carers of residents (IC)^7^ revealed strong support for using MXS in RACFs to achieve healthcare‐in‐place, concurring with the perspectives of health and aged care clinicians and managers (stakeholders).^8^, ^9^

The Medicare Benefit Schedule (MBS) lists all Australian Government health‐care services that receive a rebate under Medicare and this includes X‐ray services.^10^ Radiology services may opt to charge an out‐of‐pocket cost over and above the rebate and in that instance, residents must pay both the rebate and the out‐of‐pocket cost to the radiology service and the resident is then reimbursed the rebate by Medicare. Where residents are bulk‐billed, then the radiology service is directly reimbursed by Medicare and paid only the rebate. Additional costs are also incurred when radiology equipment is transported to the RACF, which private MXS providers have charged a ‘call‐out’ fee to residents.^11^ In November 2019, the Australian Government listed MBS item 57,541 to meet the additional ‘call‐out’ fee for radiological services for specific conditions, where the conduct of the X‐rays in the RACF could contribute to hospital avoidance. This call‐out fee rebate is applicable only when a general practitioner (GP) assesses the resident in‐person and orders an X‐ray for specific conditions including fall‐related injuries, acute abdomen or bowel obstruction, or pneumonia or heart failure.^12^

Our qualitative research found that some residents^6^ and ICs^7^ of residents were willing to pay for a ‘call‐out’ fee because of the perceived benefits to residents and ICs, though this raised equity concerns. Furthermore, one IC highlighted that the fee paid upfront was a potential barrier to residents accessing MXS.^7^ Our published research had limitations, however, in that we had not analysed data with stakeholders for economic and cost considerations. Also, of the residents interviewed, only four had used an MXS in an RACF (but not in urgent circumstances)^6^ and of the ICs interviewed, only one cared for a resident who had used an MXS (postfall).^7^ To date, there has been no other qualitative research published that has focussed on the economic and cost considerations of delivering and using MXS in RACFs.

Recruiting an additional sample of residents and ICs of residents having experienced MXS (i.e. Phase 2) and combining this new qualitative data with qualitative data from our previous research with residents, ICs and stakeholders (i.e. Phase 1), our research aimed to describe the economic and cost considerations relating to the application of MXS in RACFs, through interviews with residents, their ICs and stakeholders involved in the health care of residents.

## 2 METHODS

### 2.1 Ethics approval

The University of Adelaide Human Research Ethics Committee (HREC) provided ethics approval for the research protocol: H‐2020‐197. Stakeholders, residents and ICs provided written informed consent, except for four ICs who provided verbal informed consent (with HREC approval because of impact of COVID‐19 on RACF staff workload to obtain written informed consent).

### 2.2 Study design and setting

This study used an exploratory qualitative approach. Focus groups were planned, but due to COVID‐19 restrictions, RACF staff advised switching data collection to interviews. Therefore, one‐off semistructured interviews were conducted, in two phases. Phase 1 research protocol has been described previously but is briefly reported here.^6^, ^7^, ^8^

### 2.3 Sampling

#### 2.3.1 Both phases

In Phase 1, we approached six aged care organisations and four agreed to participate. In Phase 2, three of the four agreed to participate. Participating organisations were offered a $500 honorarium to identify an RACF site, and to assist recruiting residents and ICs and releasing staff from normal duties. Residential aged care facilities were located across metropolitan Adelaide and ranged in bed numbers (Table 1).

**TABLE 1:** Number of residents and informal carers of residents interviewed, per residential aged care facility.

	RACF (bed number range)^a^	Residents interviewed, *n*	IC interviewed, *n*
Phase 1	RACF A (151–200)^b^	5	5
	RACF B (101–150)	5	5
	RACF C (101–150)	4	6
	RACF D (50–100)	2	4
Phase 2	RACF E (151–200)	4	3
	RACF F (151–200)^b^	0	3
	RACF G (≥50)	0	1
	Total	20	27

Abbreviations: IC, informal carers; MXS, mobile X‐ray service; RACF, residential aged care facility.

^a^ Bed numbers included as a range to avoid identifying RACFs.

^b^ Same RACF.

#### 2.3.2 Phase 1

The aim was to interview representatives of multiple stakeholder groups involved in providing health care to residents in RACFs, 20 residents and 20 ICs. To recruit stakeholders, participating RACFs suggested RACF clinicians and managers and GPs that attended their RACF. Our clinical and research networks suggested clinicians with valuable clinical and management information from other stakeholder groups including paramedics, ED clinical staff, radiographers, a radiologist and a hospital avoidance program clinician. Interviewers invited potential participants via email (with an attached information sheet and consent form) to participate. Stakeholders were offered honorariums (state government health service non‐medical employees: $100; other stakeholders: $150) to participate in interviews.

For residents and ICs, the inclusion criteria included being a resident or IC (aged ≥18 years) of a resident living in a participating RACF, able to give informed consent and verbally engage in an interview, with a preference to recruit residents and ICs of residents who had experienced an MXS in the previous 12 months. There were no exclusion criteria. Residential aged care facility staff approached potential participants, gave verbal and written study information, obtained written consent and scheduled interview times. The interviewer telephoned participants to answer any questions and to schedule an interview.

#### 2.3.3 Phase 2

Residents' and ICs' recruitment was conducted following the same process for Phase 1, with the aim to interview approximately 10 residents and 10 ICs of residents who had received an MXS at the RACF within the last 12 months. Participants from Phase 1 were excluded.

### 2.4 Data collection

#### 2.4.1 Both phases

The semistructured interview guides were developed de novo for this study (Appendix S1). The stakeholder interview guide was pilot‐tested on one stakeholder. Phases 1 and 2 IC interview guides were pilot‐tested on two ICs and one IC (respectively).

Recruitment and data collection were delayed as RACF staff experienced disruption due to surges in COVID‐19 cases. Interviews were conducted face‐to‐face (31%), with stakeholders in their work setting, with residents in their shared living area or personal room and with an IC in their home. Other interviews were conducted via telephone (59%) and video conference (10%), with interviewers working from their home or work office. The interviewers (Author 1 and Author 2) were employed as researchers with PhD qualifications and extensive experience in conducting interviews and working with stakeholders and older people in health services research and practice. The interviewers did not have a prior relationship with participants, who were informed about the purpose of the interviews.

Interviews were audio‐recorded, then professionally transcribed (verbatim). Transcripts were de‐identified. Interviewers wrote field notes (including reflexive notes) after interviews. As a quality check, the interviewers reviewed each other's interview technique. The research team met frequently to discuss reflexivity, recruitment, interviews and interview schedules.

#### 2.4.2 Phase 1

Stakeholders' perspectives regarding the factors that influenced decision‐making about the use of MXS and the benefit and challenges of the call‐out MBS rebate were explored in interviews ranging from 26 to 64 minutes, conducted from 27/10/2020 to 16/02/2021, while the perspectives of residents and ICs on the use of MXS were explored in interviews ranging from 21 to 90 min, conducted from 2/11/2020 to 12/02/2021.

#### 2.4.3 Phase 2

Residents and ICs' experience of having an MXS were explored in interviews (ranging from 15 to 37 min), conducted from 22/05/2022 to 21/07/2022. Recruitment ceased in July 2022 because of an impending peak in COVID‐19 cases, which impacted on RACF staff ability to recruit participants. Data saturation was, however, achieved.

### 2.5 Data analysis

#### 2.5.1 Both phases

We took a realist epistemological approach. An iterative process of deductive and inductive analysis was driven by the research question about economic or cost considerations in providing MXS in RACFs. First, data relating to the research question was extracted from the data corpus. Thematic analysis was used, looking for repeated patterns of meaning across the data and guided by a recursive six‐phase process.^13^ This included researchers (Author 1 and Author 2) becoming familiar with the data (by reading transcripts and field notes), developing initial codes, sorting codes, developing and reviewing themes, developing a thematic map, defining and naming themes and producing the report. The researchers independently coded a subset of Phase 1 transcripts and then together reviewed coding (with the purpose of reviewing and refining coding and themes, discussing differences, amending codes and themes where necessary and confirming interpretation). This iterative process continued until all transcripts were independently coded and cross‐checked. This coding was used for Phase 2 data, which were independently coded and cross‐checked. The themes were compared between residents, ICs and stakeholders. Researchers discussed, interrogated and reviewed coding and themes several times. NVivo 12 (QSR International Pty Ltd.) was used to assist data management. Quotes are used to illustrate themes. Data relating to participant demographics and resident X‐rays were entered into SPSS 28 (IBM Corp.) and are presented descriptively.

## 3 RESULTS

Of the 27 stakeholders approached, 22 were interviewed. Of the 38 residents invited to participate, 20 were interviewed. Of the 32 ICs invited to participate, 27 were interviewed (see Appendix S2 for reasons for non‐participation).

Stakeholders included RACF managers and clinical staff (*n* = 7), GPs (*n* = 3), a hospital avoidance program clinician (*n* = 1), senior and extended care paramedics (*n* = 3), an ED consultant physician, manager and clinical nurse (*n* = 3), MXS radiographers (private and public) (*n* = 3), radiologist (*n* = 1) and an MXS manager (*n* = 1).

The mean age of residents recruited in Phases 1 and 2 was 85 years (range 72–95). Twelve residents (60%) were women. Informal carers recruited in Phases 1 and 2 included 19 (70%) daughters, four (15%) sons, three (11%) wives and one (4%) husband of residents. Of the 20 residents interviewed in Phases 1 and 2, eight (40%) had an MXS in the last 12 months. Of the 27 ICs interviewed, eight (30%) of their residents had an MXS in the last 12 months. Combining Phases 1 and 2, 16 (34%) of the residents and ICs interviewed had an MXS in the last 12 months (Table 1).

### 3.1 Themes

Four major themes were developed: (1) business considerations; (2) cost and payment process as a potential barrier to using MXS; (3) overcoming cost and payment barriers to using MXS; and (4) perceived cost benefits of MXS to the health‐care system, residents and ICs. Quotes were used to illustrate themes (Table 2).

**TABLE 2:** Themes and supporting quotes.

**Theme 1: Business considerations**
…mobile…services…have not lasted. They can't staff them and they can't make them financially viable (Stakeholder 19)
…the digital panel we put behind the patient, they're worth 50 grand …the X‐ray unit they should be using is worth about 35 grand now…the list goes on and on (Stakeholder 9)
…the costs of the van, the transportation, … radiographer's fees, your radiologist's fees (Stakeholder 19)
…we are looking at 10 X‐rays, at best, a day…that's almost a third of what a fixed site would get, at best (Stakeholder 20)
Those guys have to charge the out‐of‐pocket expenses because their overheads are horrendous (Stakeholder 14)
When we started, we did the post‐payment option where we sent out an invoice…we run into a lot of debt …we had to sit down and say ‘either we change to an upfront payment or we're just going to have to shut the business because it is not working for us’ (Stakeholder 20)
Whenever you start charging gaps, it's difficult to collect the money, so it's much more expedient to get a medical rebate from the government…if there is no gap charged and it's bulk‐billed, it's a lot easier. …I think there have been at least two mobile services started in [in another city] since the introduction of the new fee [rebate] (Stakeholder 19)
[call out rebate] … it just doesn't represent the cost input (Stakeholder 9)
**Theme 2: Cost and payment process as a potential barrier to uptake of MXS**
This is to help out the oldies, why can't the government pay the whole lot? (RACF A, Informal Carer 31, Phase 1)
…all of a sudden, they have to pay for an X‐ray. I think paying is the biggest hurdle we have because X‐rays have always been bulk billed and all of a sudden, they need to pay for it (Stakeholder 20)
No. If I have to pay it's not fair and I don't like that (RACF A, Resident 33, Phase 1)
They're entitled. They have a culture … that doesn't include paying for anything if they can get it for free (Stakeholder 8)
If I thought that it was completely satisfactory and it would save all my relatives and keep me much happier, I'd go for it, no matter what the cost (RACF C, Resident 30, Phase 1)
No [cost would not be an issue], if she needs to have it done, then we would get it done (RACF F, Informal Carer 6, Phase 2)
Well, if we had to pay $50 or $100, we'd pay it. I wouldn't be objecting to that (RACF A, Informal Carer 9, Phase 1)
It would depend if it's at a situation where dad needs just a quick check and he's not feeling well enough for transport just for an X‐ray, then the money is secondary (RACF C, Informal Carer 36, Phase 1)
So, like me, I've only got the pension and that – staying here takes nearly all of that up (RACF C, Resident 1, Phase 2)
I think it [paying for MXS] might be difficult for some people (RACF E, Resident 7, Phase 2)
…I do know there was private [MXS] service that some of the facilities had used previously, but the GPs didn't tend to use it, because it was cost prohibitive for a lot of the residents (Stakeholder 22)
We do see a lot of the lower socioeconomic facilities. We've had that feedback from families as well, ‘we couldn't afford this’ otherwise. So, yeah, I think it's being available to people who otherwise couldn't afford [to pay] (Stakeholder 10)
[to make the payment] … it's hard for me to go [to RACF], but if he [husband] works in the morning and he's home in the afternoon, I'll take the money in the afternoon when he gets home. So, I will get the money there (RACF F, Informal Carer 2, Phase 2)
We find it difficult getting a hold of the person to pay (Stakeholder 20)
**Theme 3: Overcoming cost and payment barriers to using MXS**
One of our doctors … realised that we were using [the private MXS] … and family members were struggling with the payment. … when we mentioned there's a fee involved, they would prefer just send to hospital … [GP] just said ‘no, just call this, it's free’ (Stakeholder 6)
X‐ray service will contact the family member and then they'll do the payment over the phone and then they'll [MXS] come in… But residents that don't have family members and not access to cash, we'll generally use the bulk bill service for them (Stakeholder 15)
For them [residents] to be paying a high fee for someone to come in, take an X‐ray, it's cheaper for them to go to a public hospital and wait and then get it done (Stakeholder 1)
I know that when she [mother] goes to [community radiology], she doesn't pay. She's on the pension, so she doesn't pay anything. If she has to pay for this mobile one as well, well ‘no, I'll say no’ (RACF A, Informal Carer 31, Phase 1)
…someone decides, better send them to ED because they use the Medicare and [residents] won't have to pay cash (Stakeholder 5)
…in less affluent suburbs, they [residents and informal carers]…, wouldn't go round the corner to [community radiology], they'd turn up to [public hospital] (Stakeholder 14)
…I think the new rebate has helped cover costs and helped to reduce some of the gap charges that have been previously applied (Stakeholder 19)
People need to understand that you're providing a service that's making things much more convenient for the resident, … if they're told that the government is willing to fund part of it, … I think that's more acceptable than just having a callout fee that is non‐funded. I think that will help sell the whole thing (Stakeholder 11)
I think for it to work, it would have to be an account system. … they provided the service but then you're issued – it has to be paid within seven days or whatever. (Stakeholder 01)
If that co‐payment … comes from somewhere else where the family doesn't need to worry about, that would certainly improve our response time (Stakeholder 20)
**Theme 4: Perceived cost benefits of MXS to healthcare system, residents and informal carers**
…the cost of an ambulance is around $1000… In a public ED…if you are a non‐Medicare patient…we … bill roughly about $500 … considering all up that's about $2500 of costs that we're going to incur because we didn't pay for someone to have a home X‐ray, that's ridiculous (Stakeholder 17)
Two ambulance trips in itself is $2000…let alone the care in an ED (Stakeholder 18)
[Taking mother to community X‐ray]…it means I would have to catch a taxi to [RACF]…come all the way down to [community radiology], take her all the way back because they will not put her in a taxi by herself, then come all the way back here. So cost is a big factor (RACF F, Informal Carer 2, Phase 2)
It is difficult. Firstly, getting the time [off work] to take her…. (RACF E, Informal Carer 9, Phase 2)
[daughter] would have taken me…but she's got a few health problems herself. … She lives at [town almost 70 kilometres from her mother's RACF], … so consequently it's a long way … She's got a lot of other responsibilities as well as me (RACF E, Resident 7, Phase 2)
We've got a daughter here that spends half her time on [place name, over 200ks from father's RACF]… they are quite attentive but they're still …having to keep several balls up in the air … They've got to take time out from what their other higher priorities would be (RACF E, Resident 8, Phase 2)
I couldn't afford a carer because they cost so much per hour. They're very high costs, $50 an hour or something…and they've got to be with you while you're waiting (RACF E Resident 1, Phase 2)

Abbreviations: ED, emergency department; GP, general practitioner; MXS, mobile X‐ray service; RACF, residential aged care facility.

#### 3.1.1 Theme 1: Business considerations

Private MXS providers described economic factors shaping their capacity to profitably deliver MXS. Apart from income needing to exceed costs, the X‐ray and call‐out fee rebate and payment method also shape business viability. Some stakeholders were aware of some MXS businesses that had not been viable. Business expenses (establishment and recurrent), and limits to the daily number of MXS able to be provided (through driving to appointments across a catchment area), constrained economic viability. These factors necessitated charging residents a call‐out fee, paid at the time of MXS booking (as a response to non‐payments). The call‐out fee rebate generated mixed responses. One stakeholder considered the call‐out fee rebate would help address non‐payment, particularly if a co‐payment was not charged. Radiographers in private practices, however, thought the call‐out fee rebate was insufficient to sustain their business model and charged a co‐payment to cover the gap between the call‐out fee and call‐out fee rebate.

#### 3.1.2 Theme 2: Cost and payment process as a potential barrier to using MXS

Several residents and ICs expected that MXS should be free or subsidised, to be affordable for older people. Some participants had not paid for X‐rays in the past and were reluctant or unwilling to pay for MXS. Some stakeholders, residents and ICs described an ‘entitlement mentality’, reflecting the view of others that healthcare should be free.

Some residents and ICs were willing to pay, while others indicated that they would pay for an MXS, with an upper limit. Some residents and ICs weighed issues (such as resident comfort and well‐being as well as urgency of the need for MXS) against the cost of a call‐out fee.

In some instances, participants believed they would not be able to pay very much for an MXS call‐out fee because they had limited income (usually the pension), and/or the cost of RACF meant they had little money for ‘extras’. Other residents and ICs thought that, while they could afford to pay for an MXS call‐out fee, the cost might be an obstacle for other residents. This was speculative because residents in both phases had not paid for an MXS. Some stakeholders, however, related their experience that the MXS call‐out fee had been a barrier for some residents, particularly in less‐advantaged areas.

Another cost barrier is the requirement for the co‐payment to be paid upfront, often paid by the IC. Some residents, ICs and stakeholders thought that paying the co‐payment at very short notice could be inconvenient, leading to delays in, or no, MXS provision.

#### 3.1.3 Theme 3: Overcoming cost and payment barriers to using MXS

Phase 1 data revealed strategies used to overcome cost and payment barriers by residents or ICs. Some GPs and RACF staff, for instance, used an MXS with no direct cost to residents (bulk billing or using public MXS) for residents who were unable or unwilling to pay the call‐out fee for a private MXS. Private MXS were used for those who were willing to pay and or had family to organise the payment. In other instances, stakeholders argued that in their experience, some residents who were unable to afford an MXS used other radiology services (such as ED), thereby avoiding payment. Finally, the call‐out fee rebate itself was a form of overcoming cost constraints for residents, seen as a benefit for MXS providers, residents and the sector generally.

To overcome organising unplanned upfront co‐payments, ICs and stakeholders suggested alternative payment processes. This included utilising residents' own personal funds at the RACF, or MXS providers could invoice residents (within a payment period) or invoice RACFs, who then charged the resident's existing account. To assure payment to MXS providers and RACFs, residents/ICs could have an established written payment agreement. However, some stakeholders suggested alternatives to residents making the co‐payment, such as RACFs and state health services paying for them, as well as a suggestion to increase the rebate.

#### 3.1.4 Theme 4: Perceived cost benefits of MXS to health‐care system, residents, and informal carers

Stakeholders in particular and ICs considered that the use of MXS could offset costs in other parts of the health‐care system, principally ambulance transfer and treatment in ED.

A second, less visible, cost that MXS may mitigate is the monetary and non‐monetary cost for ICs transporting and escorting residents to external X‐rays: forfeiting work (paid and unpaid) and other obligations, as well as time and transport costs, and for residents, paying for transport and an escort.

## 4 DISCUSSION

Mobile X‐ray services provided in RACFs can enable healthcare‐in‐place^6^, ^7^, ^8^, ^14^ and hospital avoidance,^5^, ^15^ benefiting residents and ICs. To the best of our knowledge, this is the only study that has addressed cost considerations for using MXS in RACFs. For example, a UK study of decision‐making about whether to manage RACF residents in‐place or transfer to ED did not indicate whether out‐of‐pocket costs for residents played a role.^16^ Similarly, hospital avoidance programs in RACFs describe using MXS, but do not mention cost considerations for residents, possibly because health services paid for MXS.^5^, ^17^, ^18^

The present study indicates for business viability, MXS providers charge residents an upfront fee (including out‐of‐pocket costs), which may impact on accessibility of MXS for all, with implications for residents, ICs and the wider health‐care system. Whilst the call‐out MBS rebate has tried to address this, enabling some MXS providers to bulk bill residents,^19^ some providers continue to charge an out‐of‐pocket cost in addition to the rebate. In our study, participants suggested an alternative to the upfront payment process, so the MXS booking was not unnecessarily delayed and MXS providers were guaranteed payment (e.g. prior agreement to use resident personal fund). Participants also suggested alternatives to residents paying (e.g. that state public health services paid). These suggestions indicate a desire for timely universal access but with a need to overcome cost barriers.

Despite participants valuing the opportunity to receive healthcare‐in‐place, our findings suggested that failure to bulk‐bill may be a barrier to some residents accessing MXS, due to unwillingness or inability to pay the upfront cost (i.e. rebate plus out‐of‐pocket costs),^6^, ^7^ reinforcing the stakeholders' observation that cost was a barrier for some residents. Out‐of‐pocket costs have been noted to be a potential or actual barrier to using health services.^20^, ^21^, ^22^

Stakeholders are supportive of residents' access to MXS within RACFs to facilitate the delivery of healthcare‐in‐place.^8^, ^9^ Some RACF staff in our study overcame residents' cost barriers by choosing MXS providers that bulk‐bill residents. Without a bulk‐billing MXS option, some residents and ICs choose public hospital radiology services, which would not generate an out‐of‐pocket expense, such as hospital transfer. Stakeholders also observed this. This negates the policy aim of the Medicare call‐out rebate of promoting healthcare‐in‐place and reducing avoidable ED presentations,^19^ and is counter to the preferences of many residents and IC to avoid transfer if possible.^6^, ^7^ Substituting ‘free’ services in response to avoiding an upfront cost with an out‐of‐pocket cost remaining to the resident after they receive their Medicare rebate has also been noted in other settings,^21^ and are unlikely to be utilised equitably among residents,^21^ many of whom have limited income with little money for ‘extras’.

The call‐out MBS rebate for MXS in Australia has the potential to have a positive national impact for residents to receive timely diagnosis and treatment, as one strategy to overcome cost constraint for residents. However, restricting MXS provider eligibility for the call‐out MBS rebate to requiring in‐person GP assessments could reduce residents' accessibility and is likely to have cost implications.^19^ If GPs cannot provide a timely in‐person assessment, which is a significant issue for many RACFs,^8^ then eligibility for the call‐out MBS rebate would not be met. This likely increases the cost for residents, who may transfer to external X‐ray facilities, where a GP referral or in‐person assessment is not required.

There are two important societal cost benefits to residents using MXS and avoiding transfer for X‐rays. First, the use of MXS may avoid the monetary (e.g. paying for taxi) and non‐monetary (e.g. forfeiting other obligations) cost implications for residents and ICs,^7^, ^14^ which are not usually accounted for in health costing.^23^ Without using MXS, the system relies on ICs' hidden input and opportunity costs, which residents prefer to avoid using.^6^ Second, participants are aware that MXS can reduce public health‐care system costs,^6^, ^7^, ^8^, ^14^, ^24^ such as ED presentation, which was the anticipated impact of the call‐out MBS rebate for common indications.^19^

### 4.1 Strengths and limitations

We developed a comprehensive understanding of the economic and cost considerations by interviewing stakeholders, residents and IC of residents, a proportion (34%) of which had experienced an MXS in the last 12 months (albeit a smaller sample than desired) from six RACF sites with a range of bed numbers. According to residents who received an MXS, they did not pay a fee, likely because GPs or RACF staff requested a bulk‐billing MXS, although it would have been helpful to know whether private or public MXS providers were used. The study was conducted within one metropolitan area serviced by private and public MXS providers, which may limit transferability of findings to areas with different service profiles.

## 5 CONCLUSIONS

Upfront co‐payments (and hence out‐of‐pocket costs) are a cost barrier for some residents, potentially reducing MXS uptake as well as leading to potentially avoidable transfers.^18^, ^21^ Furthermore, cost barriers to residents' access to MXS may negatively impact upon private MXS' financial viability through decreased demand for MXS. The Medicare call‐out rebate improves private MXS financial viability, with potential to remove the requirement for an upfront co‐payment, where the call‐out MBS rebate is sufficient. MXS providers charge a co‐payment, indicating the current rebate is insufficient to support bulk‐billing. Bulk‐billing MXS provides for residents that otherwise do not have the means to meet upfront and out‐of‐pocket co‐payment. Coexistence of private and public MXS providers may be an optimal service model. Further exploration will allow policymakers and MXS providers to understand the economic and cost considerations that will help sustain the equitable delivery of MXS in RACFs and maximise uptake, given the benefits for residents, ICs and the wider health‐care system.

## CONFLICT OF INTEREST STATEMENT

RV is the Head of Unit of Geriatric Medicine at The Queen Elizabeth Hospital in Central Adelaide Local Health Network (CALHN). RV was on the Resthaven Inc governance committee until December 2021 and previously was on the board of Resthaven Inc. RV received honorariums for presentations or expert advice from Nutricia, Abbott and Nestle in the past. RV is also co‐founder and chair of the medical advisory committee to technology start up‐ Healthyvibes.ai. GM is a surgeon in CALHN. RV and GM were not involved in participant interviews. JE was a registered general nurse working in an RACF, but interviewed participants not from organisations in which she worked. The other authors declare no conflicts of interest.

## Supporting information


Appendix S1.

Appendix S2.

## Data Availability

The data are not publicly available due to privacy or ethical restrictions. The data that support the findings of this study are available on request from the corresponding author. Requests for data should be directed to the corresponding author (joanne.dollard@adelaide.edu.au) and ensuing research will require collaboration with the chief investigators (RV and JD). Any requests will be assessed for scientific rigor by this investigator team. A request for ethics approval and/or amendment must be prepared by the requestor in line with the requirements of Human Research Ethics Committee of the University of Adelaide. This ethics amendment must be approved by the Human Research Ethics Committee of the University of Adelaide. A data sharing agreement will need to be put in place. The requestor will be responsible for providing the necessary funding required for this process, including for the provision of data.
