# Supplementary material for: Economic and cost considerations of delivering and using mobile X‐ray services in residential aged care facilities: A qualitative study
Source: Australas J Ageing. 2023 Jul 30;42(4):710–9. doi: 10.1111/ajag.13228 (PMC10947139; doi:10.1111/ajag.13228)
Supplement: Supplementary file 1 — Appendix S1. [file AJAG-42-710-s002.docx]

**Supplement material 1. Interview Schedules**

**Phase 1: Resident Interview guide**

Research objectives: To explore with residents:

1. What was important to their lives
2. Their knowledge of what mobile radiology services to RACF are, what they believe the role of such a service is as well as the value of such a service in relation to what was important to their lives
3. Their perceived benefits (including value), costs, risks, barriers and facilitators and for those who have accessed mobile X-ray their experiences of mobile X-ray services
4. Their perspective as to what the role of such a service should be (including its use for hospital avoidance and if this changes when there is ramping or with the pandemic), how important it is to them and what factors (including safety factors) ought to be considered in relation to the delivery of mobile radiology to residential aged care residents
5. Their willingness to pay for a mobile X-ray

|  | Core questions | Major prompts |
| --- | --- | --- |
| 1 | First, could you please tell me a little about yourself  Could you tell me what is important to your life now? |  |
| 2 | What do you know about mobile X-ray service?  In what ways (or how) would mobile X-rays be useful where you live?  Thinking about what you have said that is important to your life, how might mobile X-ray service help you maintain those things? | (if they don’t know, then give a brief explanation) |
| 3 | (for those who have experienced mobile X-ray)  Can you tell me about your experience in having a mobile X-ray? Can you walk me through this?  What were the pros and cons (actual or potential)  (for those who have not experienced mobile X-ray)  What do you think about mobile X-ray services being offered to residents like yourself living in an aged care home?  What would be the pros and cons for residents to have a mobile X-ray? | What are the   - Benefits and value of having mobile X-ray - Costs or burdens about having mobile X-ray - Risks or concerns about having mobile X-ray - Barriers to receiving mobile X-ray - Facilitators to receiving mobile X-ray |
| 4 | What health conditions or situations would you prefer to have a mobile X-ray?  What health conditions or situations would you prefer to go to ED for an X-ray?  What circumstances would you want to avoid going to ED?    How important is having mobile X-ray to you?    In providing a mobile X-ray service to residents in aged care homes, what factors need to be considered?  In having a mobile X-ray, what worries or concerns might you have? | - Use for hospital avoidance and if this changes when there is ramping or with pandemic - Intrinsic reasons (dementia, mobility, sensory loss, frailty, weakness) and urgency of condition - Extrinsic reasons – ramping, pandemic - Time of day and day of week   Family considerations  RACF staff being able to manage situation (workload, skills)  Waiting times for GP to attend  Waiting times for having mobile X-ray and results being provided to GP  Waiting times for treatment |
| 5 | Would you be willing to pay for mobile X-ray services so that you can have your X-ray in this aged care home?  Would your response change if it was an emergency situation?  If yes, how much would you be willing to pay? |  |
|  | If there anything else you would like to add? |  |

**Phase 1: Informal carer interview guide**

Research objectives: To explore with residents and family member:

1. What was important to their lives
2. Their knowledge of what mobile radiology services to RACF are, what they believe the role of such a service is as well as the value of such a service in relation to what was important to their lives
3. Their perceived benefits (including value), costs, risks, barriers and facilitators and for those who have accessed mobile X-ray their experiences of mobile X-ray services
4. Their perspective as to what the role of such a service should be (including its use for hospital avoidance and if this changes when there is ramping or with the pandemic), how important it is to them and what factors (including safety factors) ought to be considered in relation to the delivery of mobile radiology to residential aged care residents.
5. Their willingness to pay for a mobile X-ray

|  | Core questions | Major prompts |
| --- | --- | --- |
| 1 | First, could you please tell me a little about [name of family member]    Could you tell me what you consider is important to their life now? |  |
| 2 | What do you know about mobile X-ray service?    In what ways (or how) would mobile X-rays be useful where [name of family member] lives?    Thinking about what you have said that is important to [name of family member’s] life, how might mobile X-ray service help them to maintain those things? | (if they don’t know, then give a brief explanation) |
| 3 | (for those who have experienced mobile X-ray) Can you tell me about [name of family member’s] experience in having a mobile X-ray? Can you walk me through this?  What were the pros and cons (actual or potential)    (for those who have not experienced mobile X-ray) What do you think about mobile X-ray services being offered to residents like [name of family member] living in an aged care home?  What would be the pros and cons for residents to have a mobile X-ray? | What are the   - Benefits and value of having mobile X-ray - Costs or burdens about having mobile X-ray - Risks or concerns about having mobile X-ray - Barriers to receiving mobile X-ray - Facilitators to receiving mobile X-ray |
| 4 | What health conditions or situations would you prefer [name of family member] to have a mobile X-ray?    What health conditions or situations would you prefer [name of family member] to go to ED for an X-ray?    What circumstances would you want to avoid [name of family member] going to ED?  How important is having a mobile X-ray to you and [name of family member]?    In providing a mobile X-ray service to residents in aged care homes, what factors need to be considered?    In having a mobile X-ray, what worries or concerns might you have for [name of family member]? | - Use for hospital avoidance and if this changes when there is ramping or with pandemic - Intrinsic reasons (dementia, mobility, sensory loss, frailty, weakness) and urgency of condition - Extrinsic reasons – ramping, pandemic - Time of day and day of week        - Family considerations - RACF staff being able to manage situation (workload, skills) Waiting times for GP to attend Waiting times for having mobile X-ray and results being provided to GP Waiting times for treatment |
| 5 | Would you be willing to pay for mobile X-ray services so that [name of family member] can have their X-ray in this aged care home?  Would your response change if it was an emergency situation?  If yes, how much would you be willing to pay? |  |
|  | If there anything else you would like to add? |  |

**Phase 1 Stakeholder Interview Schedule**

Research objectives: to explore perspectives of:

1. Stakeholders about what factors influence their decision making about who should have an X-ray and when as well as where the X-ray should be performed (mobile or hospital)
2. Clinicians (RACF nurses, radiographers, paramedics, GPs) of the advantages and disadvantages of MXR services in providing care to residents in RACF in a metropolitan Australian setting
3. Stakeholders of the opportunities, expectations and challenges of implementing mobile X-ray services to residents in RACF in metropolitan Adelaide
4. Stakeholders of the usefulness and value of mobile X-rays in the care of all residents in RACF
5. Stakeholders about the benefit or challenge with the current call-out reimbursement through the MBS and suggestions as to what may improve uptake from a financial incentive perspective

|  | Core questions | Major prompts |
| --- | --- | --- |
| 1 | First, could you walk me through the process of a resident living in a RACF having an urgent type of X-ray?  What factors are considered in the decision-making about who should have an X-ray, when and where the X-ray should be performed? | - (who, where) What characteristics of residents would influence whether they would or would not be referred for mobile and hospital X-ray (intrinsic resident factors – immobility; family wishes; advance directive; likely benefit) - (who, where) What external factors influence decision making (availability of GP, pandemic, ramping, anything else) - (when) What influence does time of day and day of week have on decision making |
| 2 | What would you consider is the usefulness of MXR? | Is MXR useful for clinical diagnosis for *all* residents?  Are there some situations when MXR has not been useful?  Is MXR useful to guide management?  Would you prefer to use it more often if there was easy access to this service? |
| 3 | What are the advantages and disadvantages using MXR for providing care to residents? | - staff - residents |
| 4 | What have been (would be) the challenges of using MXR?  What of your expectations need to be met to ensure implementation of MXR is successful (and have these been met)?  What are the opportunities of a well implemented MXR service in RACF?  What are the gaps now for mobile X-ray? What else can it be used for? | Resident factors (positioning, compliance)  Staff factors (assistance)  Information flow of   - referral (what it is for, why) - results (protocols for reporting results in timely way);   Protocol of inclusion and exclusion criteria  Residents (unnecessary transfer, timely X-ray, treatment)  Staff  Conditions  Imaging modality  Urgent/non-urgent |
| 5 | What would you consider is the value of MXR? | - residents and family - RACF - clinicians - health system |
|  | The Australian government has listed an MBS item number to cover a $70 call out fee for residents living in RACFs to receive a mobile X-ray, to make it more accessible in order to avoid unnecessary transfer of frail residents to hospital emergency departments (ED) or community radiology clinics and to ensure timely diagnosis and appropriate treatment, for specific conditions:   - following a fall, - suspected pneumonia, - suspected heart failure or - acute abdominal/bowel obstruction | |
| 6 | What is the benefit with the current call out reimbursement through the MBS?  What is the challenge with the current call out reimbursement through the MBS?  What can you suggest to improve uptake from a financial incentive perspective? | - residents and family - RACF - clinicians - health system |
| 7 | Do you have any other comments, advice or recommendations in relation to mobile X-ray attending RACFs?  Was there anything else that you wanted to discuss?  Did you have any questions? |  |

**Phase 2: resident interview guide**

Research aim: To understand the experience of residents and family with the MXR service and the decision-making processes involved while accessing this service.

Research question: What was the experience of residents and family with the MXR service and the decision-making processes involved while accessing this service through RACFs?

***General introduction***

1. Prior to the interview, we would like to ask for the following information:

Age

Gender

Mobility: can’t walk; can transfer and/or walk with aid and/or assistance; can walk with aid; can walk independently

Dementia diagnosis

MXS in past 12 months, how many? Reason for your most recent MXR?

1. Introduction

Tell me about yourself?

What do you do most days? What do you enjoy?

If you enjoy going out, does anyone take you?

***Exploring the journey in detail with personal experience of the MXR service***

1. Experience

Tell me about having a MXR: what happened: from the start

| *Prompts- (get them to talk about the whole pathway, factors influencing process of decision making and information sharing between resident, family, RACF, GP, MXR service) |
| --- |
| Identifying a problem: Who first noticed there was a problem? What happened after that? |
| Making a decision to have MXR: Who suggested the use of MXR for your situation? (e.g., yourself or someone else); who was involved in helping you make a decision about having a MXR? What issues did they talk about with you; what were their reasons (e.g., COVID; avoiding ED; did you refer to an advanced care directive)? What was their involvement (family, GP, RACF staff, SAAS)? Tell me your interaction with RACF staff and the GP about the issue? Did the GP attend in person to assess you? Were you satisfied with your role in the decision-making process? |
| Impact of COVID: Tell me how COVID impacted on the process of deciding to have an X-ray (concerns re: ED/Bensons/MXR because of exposure to COVID) |
| MXR Information: Tell me about the information given to you about having the MXR? (e.g., choice between MXR providers, time, cost, follow-up). How did you get the information and what information were you given? Did you have any concerns, questions or expectations, for instance about radiation or the quality of image? Did the information you were given address these concerns? |
| MXR Cost: Did anyone discuss with you about the cost involved with the MXR service? Was your consent to pay for the cost sought? Tell me about whether cost was a factor in your decision about having an MXR? Tell me about the payment process and how well it worked for you including gap fee (out of pocket)? (Challenges and hardships about finances)? How much would you be willing to pay for access to this service? Is this a fair price for most people? |
| MXR Waiting time: How long did it take for the MXR to happen (from identifying a problem, contacting MXR service, until having MXR); what was it like waiting (tell me more; what was your experience; what were you feeling; worries); was the waiting period shorter or longer than was expected (reasons)? Were there waiting times for other things: seeing RACF staff; the GP; ambulance service? What did you do while waiting? |
| MXR Experience: Lead me through what happened, step by step. What was the experience of having a MXR like for you? (Radiographer communications skills, empathy, patience, direction with position; tell me about the people in the room; re: people in the room – what were your preferences, i.e., family there)  Was there equipment available to make the process of having the MXR easier?  Was the process of having the MXR physically comfortable?  Were there enough staff on the shift to monitor you? Did you feel safe and secure?  Did staff communicate with you?  During the whole experience did you get treatment (if needed) for: pain; other symptoms?  Had you previously heard about MXR? What did you know about it? How did you get to know about it? |
| Post MXR experience: What happened following your MXR? (GP contact, nurse contact; did anything different happen; how was that for you?)   1. For those who were transferred to hospital: What prompted your transfer to hospital; Tell me about your experience in getting to hospital (and being in ED, for example, what you were feeling when you were waiting; if they mention ’trauma’, ask what this means); 2. Comparing experience: Have you had a previous X-ray based at the hospital or a private radiology? Can you tell me about that experience in comparison to having a MXR? (e.g., How you felt, if you would have an MXR again, under what circumstances would you avoid MXR?). 3. Did you get timely feedback about the results of the MXR? Who did you get it from? Did anything else happen after you got the MXR results? |
| MXR value: What did you value about having a MXR? What would be your recommendations for the future in relation to MXR service? (Conditions applicable, use of anti-coagulants; explore other radiology techniques such as ultrasounds, CT scan, etc.) |
| Evaluation: Thinking about the whole experience of having a MXR, what would you have liked to have done differently? (Go through prompts 1 to 8). If yes, what would you have liked done differently? What worked well?  If you have had a MXR previously, how did this experience compare? Why?  Do you think your experience of staying in the RACF was better or worse than the care you would have received in an ED or hospital? |
| Potential impact of not having MXR: What would have happened if you had not had an MXR? (how could this impact on your routine? How could this impact on your family’s work or responsibilities (paid/unpaid work, caring for grandchildren)? |
| *Instruction for interviewer:  Please do not treat prompts as predetermined questions and exercise your judgement as a co-creator of data together with the interviewee.  Cormac McGrath, Per J. Palmgren & Matilda Liljedahl (2019) Twelve tips for conducting qualitative research interviews, Medical Teacher, 41:9, 1002-1006, DOI: [10.1080/0142159X.2018.1497149](https://doi.org/10.1080/0142159X.2018.1497149) |

**Phase 2: Informal carers interview schedule**

Research aim: To understand the experience of residents and family with the MXR service and the decision-making processes involved while accessing this service.

Research question: What was the experience of residents and family with the MXR service and the decision-making processes involved while accessing this service through RACFs?

***General introduction***

1. Prior to starting the interview, we would like the following information about residents:

Age

Gender

Mobility: can’t walk; can transfer and/or walk with aid and/or assistance; can walk with aid; can walk independently

Dementia diagnosis

MXS in past 12 months, how many? Reason for your most recent MXR?

1. Introduction

Tell me about yourself?

*current roles in life; social activities; commitments – volunteering; grandkids; activities have to juggle to care for Relative; what kind of things they do for/with resident*

***Exploring the journey in detail with personal experience of the MXR service***

1. Experience

Tell me about the resident having an MXR: what happened: from the start

| *Prompts- (get them to talk about the whole pathway, factors influencing process of decision making and information sharing between resident, family, RACF, GP, MXR service) |
| --- |
| Identifying a problem: Who first noticed there was a problem? What happened after that? |
| Making a decision to have MXR: Who suggested the use of MXR for the situation? (e.g., yourself or someone else); who was involved in helping you make a decision about their having an MXR? What issues did they talk about with you; what were their reasons (e.g., COVID; avoiding ED; did you refer to an advanced care directive)? What was their involvement (family, GP, RACF staff, SAAS)? Tell me your interaction with RACF staff and the GP about the issue? Did the GP attend in person to assess your family member?  Was the resident actively involved in decision-making? Were you actively involved in decision-making? |
| Impact of COVID: Tell me how COVID impacted on the process of deciding to have an X-ray (concerns re: ED/Bensons/MXR because of exposure to COVID) |
| MXR Information: Tell me about the information given to you about having the MXR? (e.g., choice between MXR providers, time, cost, follow-up). How did you get the information and what information were you given? Did you have any concerns, questions or expectations, for instance about radiation or the quality of image? Did the information you were given address these concerns? |
| MXR Cost: Did anyone discuss with you about the cost involved with the MXR service? Was your consent to pay for the cost sought? Tell me about whether cost was a factor in your decision about having an MXR? Tell me about the payment process and how well it worked for you including gap fee (out of pocket)? (Challenges and hardships about finances)? How much would you be willing to pay for access to this service for the resident; Is this a fair price for most people? |
| MXR Waiting time: How long did it take for the MXR to happen (from identifying a problem, contacting MXR service, until having MXR); what was it like waiting (tell me more; what was your experience; what were you feeling; worries); was the waiting period shorter or longer than was expected (reasons)? Were there waiting times for other things: seeing RACF staff; the GP; ambulance service? What did the resident do while waiting? What did you do? |
| MXR Experience: Lead me through what happened, step by step. What was the experience of having a MXR like for the resident? (Radiographer communications skills, empathy, patience, direction with position; tell me about the people in the room; re: people in the room – what were your preferences i.e., family there)  Was there equipment available to make the process of having the MXR easier?  Was the process of having the MXR physically comfortable for the resident?  Were enough staff on the shift on to monitor the resident? Did the resident feel safe and secure?  Did staff communicate with you?  During the whole experience did the resident get treatment (if needed) for: pain; other symptoms  Had you previously heard about MXR? What did you know about it? How did you get to know about it? |
| Post MXR experience: What happened following the MXR? (GP contact, nurse contact; did anything different happen; how was that for you)   1. For those who were transferred to hospital: What prompted the transfer to hospital; Tell me about the experience of getting to hospital (and being in ED- for example-what you were feeling when you were waiting; if they mention ’trauma’, ask what this means); 2. Comparing experience: Have they had a previous X-ray based at the hospital or a private radiology? Can you tell me about that experience in comparison to having an MXR? (e.g., How they felt, if they would have an MXR again, in what circumstances would you avoid MXR?). 3. Did you get timely feedback about the results of the MXR? Who did you get it from? Did anything else happen after you got the MXR results? |
| MXR value: What did you value about the resident having a MXR? What would be your recommendations for the future in relation to MXR service? (Conditions applicable, use of anti-coagulants; explore other radiology techniques such as ultrasounds, CT scan, etc.) |
| Evaluation: Thinking about the whole experience of having a MXR, what would you have liked to have been done differently? (Go through prompts 1 to 8). If yes, what would you have liked done differently? What worked well?  If the resident has had a MXR previously, how did this experience compare? Why?  Were you with the resident during the MXS? What influenced this decision?  Do you think the resident’s experience of staying in the RACF was better or worse than the care they would have received in an ED or hospital? |
| Potential impact of not having MXR: What would have happened if the resident had not had an MXR? How could this impact on your routine work or responsibilities (paid/unpaid work, caring for grandchildren)? |
| *Instruction for interviewer:  Please do not treat prompts as predetermined questions and exercise your judgement as a co-creator of data together with the interviewee.  Cormac McGrath, Per J. Palmgren & Matilda Liljedahl (2019) Twelve tips for conducting qualitative research interviews, Medical Teacher, 41:9, 1002-1006, DOI: [10.1080/0142159X.2018.1497149](https://doi.org/10.1080/0142159X.2018.1497149) |
