# Supplementary material for: Economic and cost considerations of delivering and using mobile X‐ray services in residential aged care facilities: A qualitative study
Source: Australas J Ageing. 2023 Jul 30;42(4):710–9. doi: 10.1111/ajag.13228 (PMC10947139; doi:10.1111/ajag.13228)
Supplement: Supplementary file 2 — Appendix S2. [file AJAG-42-710-s001.docx]

Supplementary material 2

Table. Reason for non-participation.

| Reason for non-participation | Stakeholders | Residents | Informal carers |
| --- | --- | --- | --- |
| Declined (did not feel well, wanted family present; recently participated in research, not interested, family declined, no reason given) | 1 | 10 | 1 |
| Unable to be contacted, did not respond or indicated they lacked time | 3 | 2 | 3 |
| Did not remember having an MXS | 0 | 3 | 0 |
| Unaware their family member had received an MXS | 0 | 0 | 1 |
| Withdrew (unwell, no reason). | 1 | 3 | 0 |
